# Optimization of Hole-Flanging by Single Point Incremental Forming in Two Stages

**DOI:** 10.3390/ma11102029

**Published:** 2018-10-18

**Authors:** Domingo Morales-Palma, Marcos Borrego, Andrés J. Martínez-Donaire, Gabriel Centeno, Carpóforo Vallellano

**Affiliations:** Department of Mechanical Engineering and Manufacturing, Universidad de Sevilla, Camino de los Descubrimientos s/n, 41092 Sevilla, Spain; mborrego@us.es (M.B.); ajmd@us.es (A.J.M.-D.); gaceba@us.es (G.C.); carpofor@us.es (C.V.)

**Keywords:** sheet metal forming, flanging, hole-flanging, incremental sheet forming, Single Point Incremental Forming (SPIF), finite element analysis, thickness distribution

## Abstract

Single point incremental forming (SPIF) has been demonstrated to accomplish current trends and requirements in industry. Recent studies have applied this technology to hole-flanging by performing different forming strategies using one or multiple stages. In this work, an optimization procedure is proposed to balance fabrication time and thickness distribution along the produced flange in a two-stage variant. A detailed analytical, numerical and experimental investigation is carried out to provide, evaluate and corroborate the optimal strategy. The methodology begins by analysing the single-stage process to understand the deformation and failure mechanisms. Accordingly, a parametric two-stage SPIF strategy is proposed and evaluated by an explicit Finite Element Analysis to find the optimal parameters. The study is focused on AA7075-O sheets with different pre-cut hole diameters and considering a variety of forming tool radii. The study exposes the relevant role of the tool radius in finding the optimal hole-flanging process by the proposed two-stage SPIF.

## 1. Introduction

Single point incremental forming (SPIF) is the simplest process variety within the incremental sheet forming (ISF) technologies. These technologies, with their germ in the late 1960’s of last century (as reported in [1]) and with a strong development since the 2000’s [2], have entirely proved to accomplish the current trends and requirements in industry including rapid, flexible, economic as well as environmental friendly manufacturing.

Besides, ISF processes applied to sheet metals present an additional advantage compared to conventional processes, consisting of an enhancement of formability [3,4] well above the Forming Limit Curve (FLC) for necking, allowing to deform the sheet until the onset of ductile fracture represented in the material Forming Limit Diagram (FLD) by means of the Fracture Forming Limit (FFL) in the case of in-plane tension corresponding to mode I of fracture mechanics. This benefit of ISF processes in terms of formability has permitted the application of SPIF (and other ISF varieties) to the manufacturing of geometrically complex and demanding components, such as automotive [5], aerospace [6] or even biomedical devices [7], as well as the production of specific shapes into an industrial part, as it is the case of flanges manufactured by incremental forming [8].

Regarding the production of hole flanges by means of incremental forming processes, the process consists of a sheet blank with an initial pre-cut hole which is progressively deformed by the forming tool, usually carried out in a number of steps or stages. Indeed, in this usual case of multi-stage hole-flanging by SPIF, the definition of the strategy is of paramount importance in order to optimize formability and/or other process outputs. Different multi-stage strategies have been proposed since 2010 in a series of research works such as [9,10]. Nevertheless, all the strategies presented so far including multiple stages present an obvious and not easily resolvable drawback: they are very time consuming.

With the aim of minimizing the process time attained by multi-stage strategies in single point incremental forming, the authors has recently proposed the use of a single-stage process for the manufacturing of hole flanges by SPIF [11]. This analysis of the single-stage variant has demonstrated its capability for the production of functional hole flanges with a remarkable reduction of the process duration. However, the results obtained in terms of thickness distribution along the resulting flange have to be definitely improved.

In this scientific and technological framework, the authors have recently proposed in two preliminary numerical investigations [12,13] the adoption of an alternative two-stage variant for the design of the hole-flanging process by SPIF. Indeed, this two-stage variant can be seen as a balance between the very time consuming of multi-stage strategies and the poor results in terms of thickness distribution provided by single-stage SPIF, allowing to produce hole flanges with homogeneous thickness in a relative short process time.

In this context, this paper presents a detailed numerical and experimental investigation with the overall objective of providing an optimal two-stage strategy in terms of thickness distribution for the hole-flanging process by SPIF. With this purpose, the deformation mechanism along the hole-flanging process by SPIF is analytically evaluated under the assumption of membrane stresses. This evaluation allows understanding the factors affecting the thickness distribution in the single-stage variant and permitted to propose and evaluate different strategies for the tool trajectory corresponding to the first stage. After selecting the most adequate strategy, the geometrical parameters defining the first stage are numerically optimized by launching an established Finite Element (FE) numerical procedure with Abaqus^®^/Explicit. The FE model makes use of a series of scripts in Python^®^ for automating the tool path definitions and the whole optimization process. Finally, the numerical results after the first and second stages are compared with the corresponding experimental thickness distributions for the case of the optimal hole-flange.

This research work has improved the current state of the art in the hole-flanging process by SPIF with the following contributions: (i) proposing and corroborating experimentally the two-stage variant of the hole-flanging process by SPIF as a balance compared to the very time consuming multi-stage strategies proposed so far and the single-stage process recently proposed by the authors; (ii) presenting the most adequate parametric two-stage strategy that was schematically defined after identifying analytically the deformation mechanism during the single-stage variant; and (iii) establishing an experimentally validated and fully automated numerical procedure that permits an optimization of the process parameters for obtaining a satisfactory thickness distribution along the flange after only two forming stages.

## 2. Methodology

The proposed methodology consists in analysing the process deformation and the flange thickness of the single-stage SPIF process with the aim of establishing a new SPIF strategy to homogenise the thickness profile along the flange.

### 2.1. Experimental Background: Hole-Flanging by SPIF in a Single Stage

In an above cited previous work [11], the authors developed an experimental study consisting on the analysis of the physical mechanisms involved in the single-stage hole-flanging process by SPIF, aiming to evaluate the mechanical conditions upon which sheet failure takes place. With this purpose, the proposed single-stage strategy was utilized to perform a series of experimental tests on AA7075-O metal sheets of initial thickness $t_{0}=1.6$ mm using different forming tools. As a result, the maximum flange that can be successfully achieved was manufactured and analysed.

**Figure 1:**
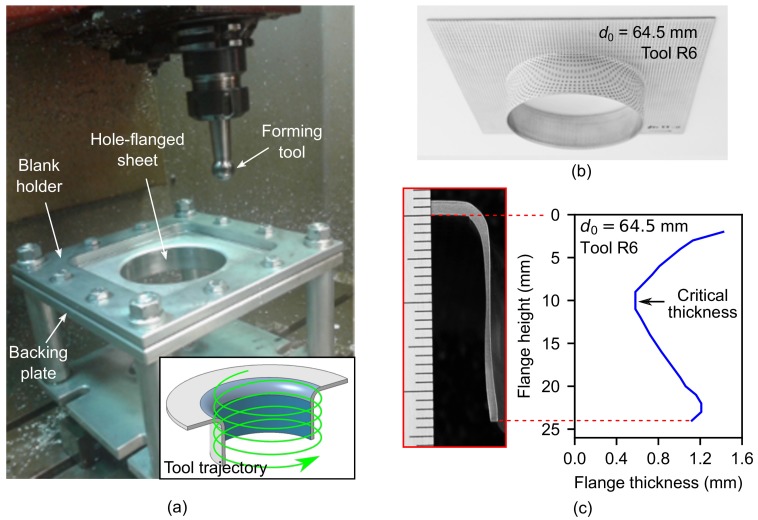
Hole-flanging by Single point incremental forming (SPIF) in a single stage of an AA7075-O sheet blank of 1.6-mm thickness: (**a**) experimental setup; (**b**) hole-flanged sheet part; and (**c**) thickness distribution along the flange.

The experimental tests were carried out on a 3-axis milling CNC machine VMC-200 (EMCO, Lingen, Germany). The experimental setup and a schema of the helical tool trajectory is shown in Figure 1a. A blank holder and a backing plate with a 100-mm diameter circular hole were used to fix the sheet blanks to the machine table. Specimens were electro etched with a circle grid pattern at the outer sheet surface to analyse strains. Three different hemispherical tool radii were used (R=6, 8 and 10 mm) to analyse the bending effect (measured by the sheet thickness to tool radius ratio, t/R) on formability. The feed rate was set to 1000 mm/min and 2 spindle speed were used, 0 rpm (locked tool) and 1000 rpm clockwise. A step-down of 0.2 mm/rev was used for the helical trajectories. A special lubricant for metal forming applications, Iloform TDN81 (Castrol, Liverpool, UK) was used to minimise friction. The final flange height, thickness profile along the flanges and surface roughness were analysed on the final parts.

Sheet blanks with different pre-cut hole diameter $d_{0}$ ranged from 55 to 82 mm were progressively deformed until producing hole flanges with a final inner diameter of $d_{f}=95.8$ mm. Figure 1b shows a sample of successful hole-flanged sheet. The limiting values of $d_{0}$ to define the limit forming ratio ($LFR=d_{0}/d_{f}$) and obtain successful specimens were 64.5, 61.0 and 57.5 mm for tool radii of 6, 8 and 10 mm, respectively.

Some successful and failed specimens were cut for measuring the thickness in the profile view and, in case of fractured specimens, to validate the previous measurements along the crack. Figure 1c shows the thickness profile of a successful hole-flanged sheet. As can be observed, there are 3 distinguishable zones along the flange, from top to bottom: (1) a zone near the flat undeformed sheet, whose thickness is progressively decreasing; (2) an intermediate or critical zone, where the thickness is the smallest and the sheet failure tends to occur; and (3) the edge zone, where thickness is progressively increasing.

### 2.2. Analysis of the SPIF Process Deformation

**Figure 2:**
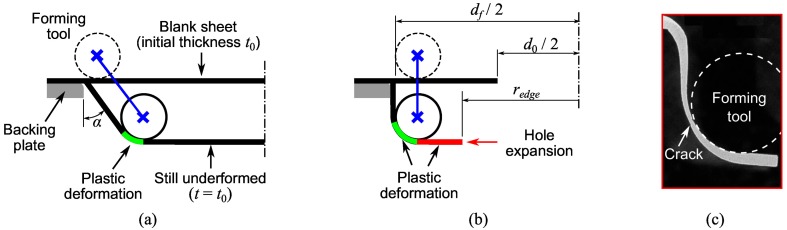
Schema of axisymmetric part deformation by SPIF of (**a**) inclined-wall part and (**b**) hole-flanged part; and (**c**) fractography of the failure zone for an interrupted hole-flanging test [11].

In previous work, the stress/strain states accomplished in sheet metal deformed by SPIF were evaluated by means of membrane analysis [14,15,16]. The analytical model provides insight to the fundamentals behind the enhanced material formability of the SPIF process. The membrane analysis in axisymmetric components, illustrated in Figure 2a, was used to explain aspects of the process deformation and formability. As a result, it was pointed out that the meridional stress component ($\sigma_{\phi }$) is mainly responsible of sheet thinning and opening of cracks (mode I of fracture mechanics) at the transition zone between the inclined wall and the corner radius of the sheet, in contact with the forming tool. These conclusions seems to be also valid for hole-flanging operations, represented schematically in Figure 2b. Indeed, the authors observed in failed specimens by single-stage SPIF that fracture initiates at the wall-tool interface limit [11], as can be seen in Figure 2c. Note that the onset of the crack coincided with the greatest thickness reduction or critical thickness.

The membrane analysis for SPIF of inclined-wall parts shows that the meridional stress $\sigma_{\phi }$ increases and sheet thickness *t* decreases along the sheet-tool interface, from the formed wall to the still undeformed zone [15]. This qualitative description of the thickness distribution agrees very well with experimental observations in hole-flanging by single-stage SPIF, as depicted in Figure 2c. The membrane theory also predicts well the influence of the bending ratio t/R on the material formability of hole-flanging components [11].

On the other hand, hole-flanging has some particularities with respect to the conventional testing geometries in SPIF (usually cones and pyramids) that were previously analysed by the membrane theory. The main difference of both deformation processes illustrated in Figure 2a,b relies on the wall angle α, as well as in the circular hole of the sheet blank. In a SPIF process with a low or moderate α value, the plastic deformation is localised in the small zone in contact with the tool. Thus, the sheet thickness of the inclined-wall part can be estimated by the sine law, $t=t_{0}\;\sin\alpha$, while the still flat material has not been plastically deformed yet and remains its initial thickness $t_{0}$. On the other hand, SPIF of components with severe α values up to 90° increase considerably the meridional stress distribution along the wall. In this situation, the plastic deformation extends beyond the area of contact with the forming tool, as the one that produces the hole expansion in hole-flanging (see schema in Figure 2b).

The hole expansion leads a plastic deformation of material that is not in contact with the forming tool. The resistance of this material zone during its radial and circumferential stretching and its effect on the forming tool action is of great importance to understand the deformation process.

**Figure 3:**
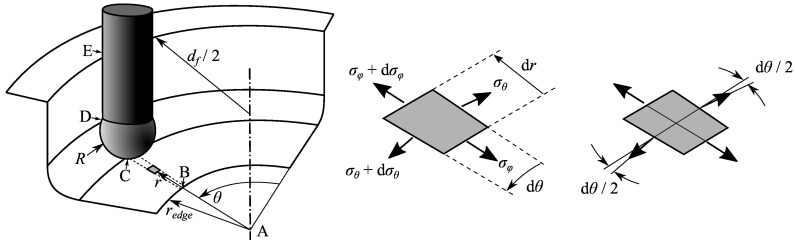
Membrane analysis of hole-flanging by SPIF in a single stage: schematic representation of a shell element located in the still flat zone under circumferential and radial stretching and details showing the acting stresses.

Figure 3 shows a forming tool acting on a sheet during hole-flanging by SPIF in a single stage. The following analysis is based on the one developed by Silva et al. [15] for axisymmetric inclined-wall parts. The analysis focuses on a shell element located in the material being radially and circumferentially stretched due to the hole expansion and in the influence zone of the forming tool. Because of axial symmetry, principal stresses are assumed to be the circumferential ($\sigma_{\theta }$), meridional ($\sigma_{\phi }$), and thickness ($\sigma_{t}$) stresses.

In Figure 3, the tool is positioned along the sheet section A-B-C-D-E and the shell element is in the flat zone adjacent to the hole edge, between points B and C. The shell element is located at coordinates (*r*, θ) and has a thickness $t<t_{0}$ due to the radial and circumferential expansion. The stresses acting on the shell element are also shown. Resolving the force equilibrium along the meridional direction one obtains

(1) $$\sigma_{\phi }\;t\;r\;d\theta +\sigma_{\theta }\;t\;dr+\left(\sigma_{\theta }+d\sigma_{\theta }\right)\;t\;dr\;\sin\left(d\theta /2\right)=\left(\sigma_{\phi }+d\sigma_{\phi }\right)\;t\;\left(r+dr\right)d\theta$$

After neglecting higher order terms and simplifying the above equation by taking into account that sin(dθ/2)≈dθ/2, Equation (1) results in

(2) $$\sigma_{\theta }\;dr=\sigma_{\phi }\;dr+r\;d\sigma_{\phi }\;\to \;\frac{d\sigma_{\phi }}{dr}=\frac{\sigma_{\theta }-\sigma_{\phi }}{r}.$$

The principal stresses acting on the shell element can be identified as

(3) $$\sigma_{1}=\sigma_{\theta }>0\;,\;\sigma_{2}=\sigma_{\phi }>0\;,\;\sigma_{3}=0$$

According to the Tresca yield criterion, the term $\sigma_{1}-\sigma_{3}$ should be equal to the yield stress $\sigma_{Y}$. Inserting in Equation (3) one obtains $\sigma_{Y}=\sigma_{\theta }$. Substituting in Equation (2) and integrating, the meridional stress in the shell element is found as

(4) $$\sigma_{\phi }=\sigma_{Y}\left(1-\frac{r_{B}}{r}\right)$$

where the boundary condition $\sigma_{\phi }=0$ at the hole edge ($r=r_{B}$) was used to find the integration constant. Equation (4) allows to evaluate the meridional stress $\sigma_{\phi ,C}$ in the transition zone with the corner radius of the sheet in contact with the tool ($r=r_{C}=d_{f}/2-R$).

According to Silva et al. [15], the reason why thinning occurs in the corner radius C-D has to do with $\sigma_{\phi }$ acting in that zone. Indeed, thinning in C-D tends to balance the increment of meridional stress so that $t\cdot \sigma_{\phi }$ remains constant. According to this expression, thinning is especially relevant at point D. Besides, given that thickness at point C decreases as the SPIF process evolves and the hole expands, the sheet thickness in point D is getting more and more thin, in agreement with the experimental observations [11].

Therefore, the sheet thinning at point D (critical thickness along the flange) could be diminished during the SPIF process by controlling the resistance of the material being radially and circumferentially stretched. According to Equation (4), this could be achieved by reducing the yield stress or by increasing the hole edge radius $r_{B}$. This later solution can be achieved by performing a previous SPIF stage to modify the part geometry in the zone B-C.

### 2.3. Proposal for an Improved SPIF Strategy

**Figure 4:**
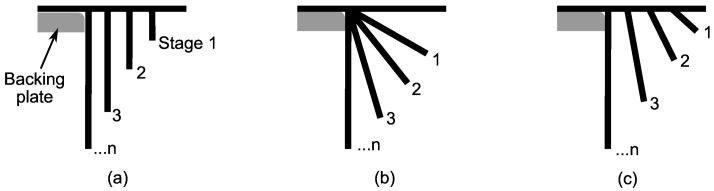
Schematic representation of hole-flanged sheet sections produced by SPIF using different multi-stage strategies producing successive (**a**) concentric cylinders; (**b**) conical frustums with increasing angle; and (**c**) conical frustums with increasing angle and major diameter.

Figure 4 presents 3 multi-stage SPIF strategies for hole-flanging proposed by Cui and Gao [9] to analyse the thickness distribution along the sheet flange of AA6010 sheets. Strategies consist of producing one or more simple intermediate part shapes, and the final hole-flanged part, by programming linear sections. Experimental tests were performed using a number of stages ranged from 2 to 5. Different combinations of increasing values for wall angle and part diameter were used. In a later work, Bambach et al. [10] used similar strategies to evaluate and exploit the flexibility of the SPIF technology to perform hole-flanging of mild steel sheets. Both experimental works concluded that flange thickness distribution obtained with the strategy illustrated in Figure 4a was the most uniform among the forming strategies considered. However, this strategy produced actually a lower thinning in the initial part of the flange compared to the lower and more homogeneous thickness as approaching to the edge.

**Figure 5:**
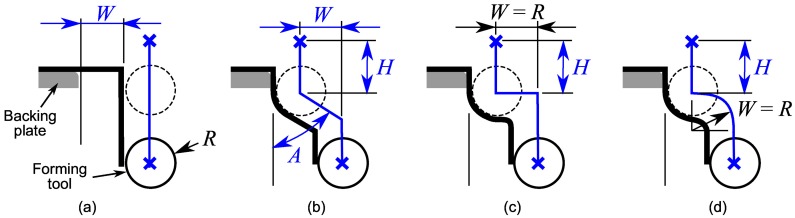
Schema of the first stage in hole-flanging by two-stage SPIF: (**a**) straight section due to Cui and Gao [9]; (**b**) 3-parameter section analysed in [12]; (**c**) prototype based on the three-parameter section; and (**d**) proposed section.

Consequently, the first strategy proposed to perform hole-flanging by SPIF in two stages consists in programming two concentric helical paths. Figure 5a represents the tool path along the theoretical straight part section that defines the first stage. The distance *W* defines the hole diameter of the intermediate sheet part. As exposed in Section 4.3, this two-stage strategy produces a final thickness distribution of the flange according to the observations of the studies mentioned above [9,10]. Thus, an alternative strategy is needed to further reduce the thickness at the initial part of the flange.

Regarding the analysis of the single-stage SPIF process presented in the previous section, the sheet thickness distribution could be better homogenised by modifying the part geometry adjacent to the hole edge (zone B-C in Figure 3). This can be done by performing a previous hole-flanging operation localised in that zone to increase the hole diameter, as illustrated in Figure 5b–d. The main difference with the previous strategy is that the operations start as the single-stage SPIF process and the tool trajectories are later modified to reduce the pressure with the forming tool in the critical zone of the sheet.

The SPIF strategy shown in Figure 5b was analysed in a recent numerical study [12]. An automated procedure was performed to simulate the flange thickness using Abaqus/Explicit. A series of 18 sets of consistent values of *W*, *A* and *H* were chosen to perform the study. It was concluded that the most homogeneous flange thickness was obtained using the highest angle value (A=80° in the study) and a distance *W* equals to the tool radius *R*.

In the present work a SPIF strategy is proposed by taking into account the conclusions of the previous study. Thus, the angle and width parameters are fixed to A=90° and W=R, as represented in Figure 5c. It should be noted that the horizontal transition movement in the case of A=90° requires an appropriate trajectory to avoid unexpected collisions due to the material springback. For instance, a spiral path could be used to smooth the sheet surface. Another option is to use a retract rapid movement upwards and inwards the hole and start the second drop from above. The optimum value of the height *H* is assumed to be dependent on the material and process geometry.

The above strategy can be further improved by programming a faster circular transition movement instead of the horizontal one, as represented in Figure 5d. Indeed, simulations performed with both options have shown almost identical thickness distributions. Therefore, in this work only the results obtained with strategies (a) and (d) shown in Figure 5 are analysed and compared.

## 3. Numerical Model

In order to evaluate the proposed strategy, numerical simulations have been performed using the commercial finite element code Abaqus/Explicit. The FE model has been parametrised to reproduce the experimental tests with different tool radii *R* and pre-cut hole diameters $d_{0}$ of the sheet blank developed in previous work [11].

**Figure 6:**
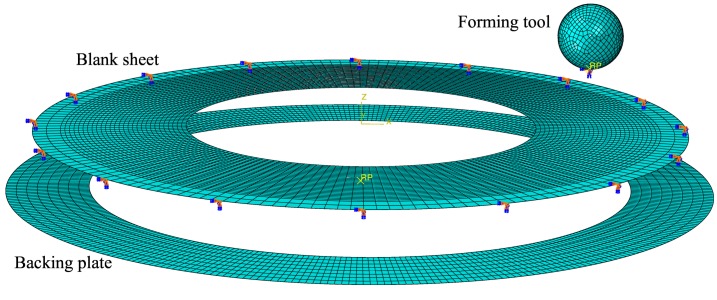
FE model including the meshed pre-cut sheet, backing plate and forming tool.

Figure 6 shows the FE model. The sheet blank was modelled as a deformable circular ring of 2D-shell elements with five integration points through the thickness. All degrees of freedom of the nodes located at the outer edge of the blank were fixed to reproduce the blank holding. The backing plate and the forming tool were modelled as discrete rigid surfaces. The former was modelled as a circular ring of 100-mm inner diameter and the latter as a sphere. The material was 7075-O aluminium alloy sheet of 1.6 mm thickness. The mechanical properties of the material are summarized in Table 1. The von Mises yield criterion and the Hollomon type law were used to describe the plasticity behaviour and hardening, respectively.

It should be noticed that the assumption of material isotropy aims to reduce the complexity of the FE model and accelerate the simulations. In previous work [11], the values of Lankford’s anisotropy coefficients obtained experimentally ($r_{0}$, $r_{45}$, $r_{90}$) did not differ strongly from an isotropic behaviour. On the other hand, some recent research works [17,18] have confirmed that the use of an anisotropic yield criterion is not a determinant factor for predicting the final geometry of axisymmetric conical testing parts deformed by SPIF.

The sheet blank was meshed with 180 nodes around the circumference and equispaced every 0.8 mm approximately along the radial direction. The total number of elements ranged from 7380 to 8100 for the maximum and minimum values of $d_{0}$ used in simulations: 64.5 and 57.5 mm, respectively. A *surface-to-surface* contact algorithm with the finite sliding formulation was used. A friction coefficient of 0.1 was assumed.

**Figure 7:**
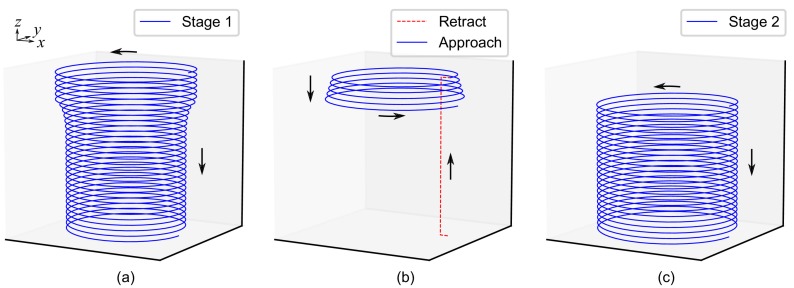
Schema of the tool tip trajectory for the proposed hole-flanging by SPIF in two stages: (**a**) first stage; (**b**) transition movement and (**c**) second stage.

The tool trajectories were generated by programming Python scripts to locate precisely the tool tip coordinates. A simple helix was used to simulate the hole-flanging process by SPIF in a single stage. As can be observed in Figure 7a, the tool tip trajectory for the stage 1 of the 2 proposed process reproduces the sheet geometry of the intermediate part (see Figure 5d). A *retract and approach* transition movement was modelled to link both forming stages. Note in Figure 7b that the approach movement consists in a helical path with increasing diameter to take into account the springback of the metal sheet. In stage 2, the forming tool continues from the position it was separated from the wall at the end of the first stage.

According to the experimental tests in [11], the tool step-down was set to 0.2 mm/rev. As in previous work [12], mass scaling technique was used to speed up te computing time. Every single simulation required around 6–8 h in a 64-bit PC with an Intel Core i5 processor and 8 GB of RAM.

## 4. Results and Discussion

### 4.1. Validation of the FE Model

**Figure 8:**
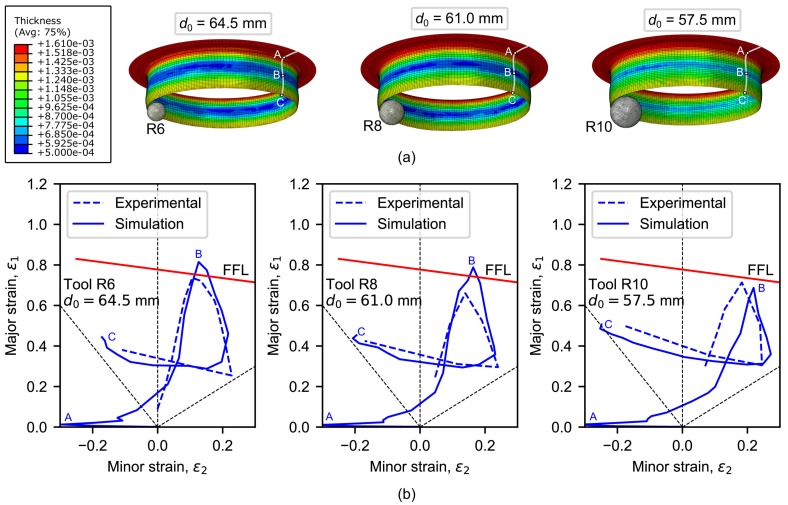
Simulations of hole-flanging processes by single-stage SPIF and comparison with experiments performed in [11]: (**a**) thickness distribution on 3D views and (**b**) FLD showing strain distributions along the flange.

As said in Section 2.1, the limiting values of pre-cut hole diameter 64.5, 61.0 and 57.5 mm were experimentally determined in a previous work to obtain successful hole-flanged specimens by single-stage SPIF using tool radii of 6, 8 and 10 mm, respectively [11]. These 3 experiments have been simulated to validate the prediction capabilities of the FE model. Figure 8a presents the sheet thickness distribution on 3D views. The section *A-B-C* along the flange represents the zone near the flat undeformed sheet (point *A*), the critical zone (point *B*) and the flange edge (point *C*). As can be seen, a higher thickness reduction in the intermediate zone along the flange is shown, in well agreement with the experimental results.

**Figure 9:**
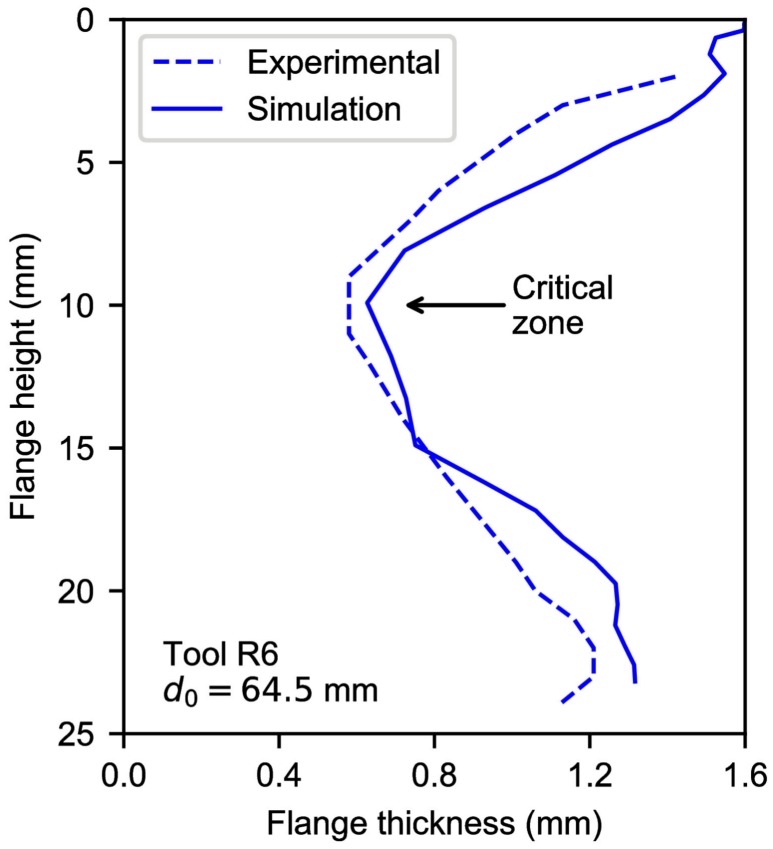
Thickness distribution along the flange of a hole-flanging process by single-stage SPIF: simulation and experiment [11].

Figure 8b depicts the principal strains measured at the outer sheet surface along the section *A-B-C* of the flange in a FLD for experiments vs. simulations. The FFL curve which was determined in previous work [11] is also represented. As can be seen, the numerical strain evolutions reproduces successfully the experimental trend, describing a loop, beginning from the top of the flange, reaching a maximum value around the critical zone and ending at the flange edge. The thickness distribution for the experimental test using the 6-mm tool radius is compared with that obtained by the FE model in Figure 9. In general, the thickness predictions of the FE model are quite fair.

### 4.2. Analysis of Flange Deformation by Single-Stage SPIF

**Figure 10:**
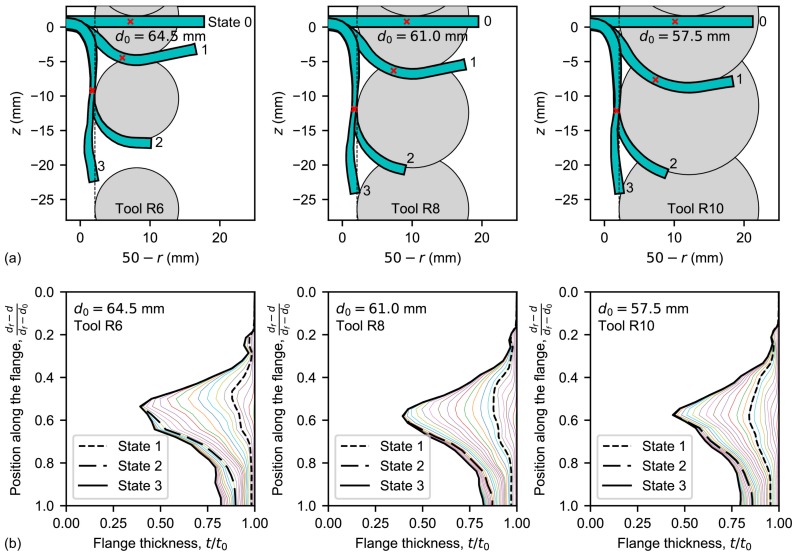
Simulations of hole-flanging processes by single-stage SPIF: (**a**) flange profile and tool position; and (**b**) flange thinning.

Figure 10a shows the thickness profile evolution and the tool position for the simulated single-stage SPIF processes. Four simulation states are depicted: (0) the initial state; (1) a step-down equal to the tool radius; (2) the tool position when the minimum thickness is reached; and (3) the final state. A red cross indicates the critical position, that is, where the maximum thinning will occur in the flange.

At the beginning of the SPIF process, forming tool sinks into the sheet and the contact area increases. In states 1 to 2 (see Figure 10a), the above exposed analytical framework for SPIF can be used to describe the process deformation. Indeed, the most pronounced thinning occurs in the formed flange closest to the contact with the tool for all the simulations, as can be observed in state 2 of Figure 10a. At the end of the process, the material from the critical thickness point to the hole edge seems at first glance to bend without thinning. According to the analytical framework, the reason is that the edge has expanded almost to the end and, thus, this material zone offers much less resistance in the meridional direction. Notice that the length of this region seems to be independent of the tool radius.

The flange thinning evolution obtained from simulations above is presented in Figure 10b. The successive thickness curves have been evaluated every 1 mm of step-down increment. As can be seen, the minimum thickness position moves upwards along the flange while the tool is sinking into the sheet (until state 1). Then, it moves down from state 1 until reach the critical thickness at state 2 according to the analytical framework presented above. Notice that final *bending* of the edge zone (states 2 to 3) produces a slight progressive thinning localised in that zone due to the hole expansion.

### 4.3. Analysis of the Flange Deformation in the Two-Stage SPIF Process

**Figure 11:**
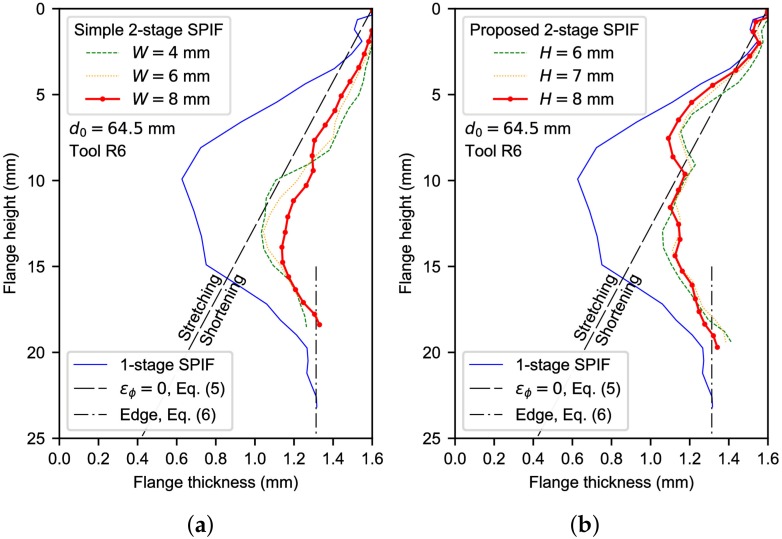
Flange thickness distribution by two-stage SPIF: (**a**) straight section and (**b**) proposed strategies.

Figure 11 depicts the thickness distributions of the flange from FE simulations using a 64.5-mm pre-cut hole diameter and a 6-mm tool radius. Thickness curves correspond to the straight section and proposed strategies by two-stage SPIF illustrated in Figure 5a,d, respectively. The former has been evaluated by setting W=6, 7 and 8 mm. According to the conclusions of Cui and Gao [9], it is expected that these parameter values are closed to the optimal value. On the other hand, a simple choice H=R=6 mm based on experimental observations has been used for selecting the parameter value of the proposed two-stage strategy. Besides, H=7 and 8 mm have also been evaluated for comparison. The thickness profile for the hole-flanging process by single-stage SPIF is also represented.

As can be seen, the two-stage SPIF processes decrease the thickness reduction significantly, about a 40–50% in average, compared to the single-stage process. The straight section strategy (see Figure 11a) yields a similar shape of the thickness profile to the single-stage process: a progressive thinning along the top of the flange, a minimum thickness in the midway (around a flange height of 13–15 mm) and a progressive thickening in the edge zone. This description agrees well with experimental observations by Cui and Gao [9] for AA6010 sheets.

The proposed two-stage SPIF process (see Figure 11b) produces a more pronounced flange thinning in the top zone of the flange compared to the two-stage SPIF process using the straight section. This is attributed to the edge resistance to be circumferentially and radially expanded. Such difference can be further analysed by evaluating flange stretching in meridional direction, $\varepsilon_{\phi }$. Thus, the thickness distribution of a hypothetical hole-flanging operation where the material is not strained in this direction can be found as

(5) $$\varepsilon_{\theta }:\varepsilon_{t}:\varepsilon_{\phi }=1:-1:0\;\to \;t\left(r\right)=t_{0}\cdot \frac{2\;r}{d_{f}}$$

where *r* is the radial coordinate. This linear function is also depicted in Figure 11 to distinguish stretched and shortened material along the flange. As can be observed, thickness distributions of the two-stage SPIF using the straight section indicate a shortened flange. Instead, stretching occurs in both single-stage SPIF and the proposed two-stage SPIF in the top zone of the flange.

Figure 11b reveals that material stretching produced by the proposed two-stage SPIF led to a more uniform thickness in the intermediate part of the flange than the straight section strategy, around 1.1–1.2 mm thickness. The higher the *H* value, the higher thinning in the top zone (around 7-mm flange height) and the lower thinning in the bottom zone (around 13-mm flange height). The optimum *H* value that seems to homogenise flange thickness is 8 mm. Nevertheless, given that thickness differences are less than 10%, the simplest choice of H=R has been assumed.

It should be noticed that edge thinning in hole-flanging processes is controlled by pure tension conditions and, therefore, it can not be modified by any forming tool. In this situation, the final thickness at the hole edge can be predicted as

(6) $$\varepsilon_{\theta ,edge}:\varepsilon_{t,edge}:\varepsilon_{\phi ,edge}=1:-0.5:-0.5\;\to \;t_{edge}=t_{0}\cdot \sqrt{\frac{d_{0}}{d_{f}}}$$

This expression is represented in Figure 11a,b as vertical lines. As can be seen, it reproduces very well the edge thickness predicted by SPIF simulations.

**Figure 12:**
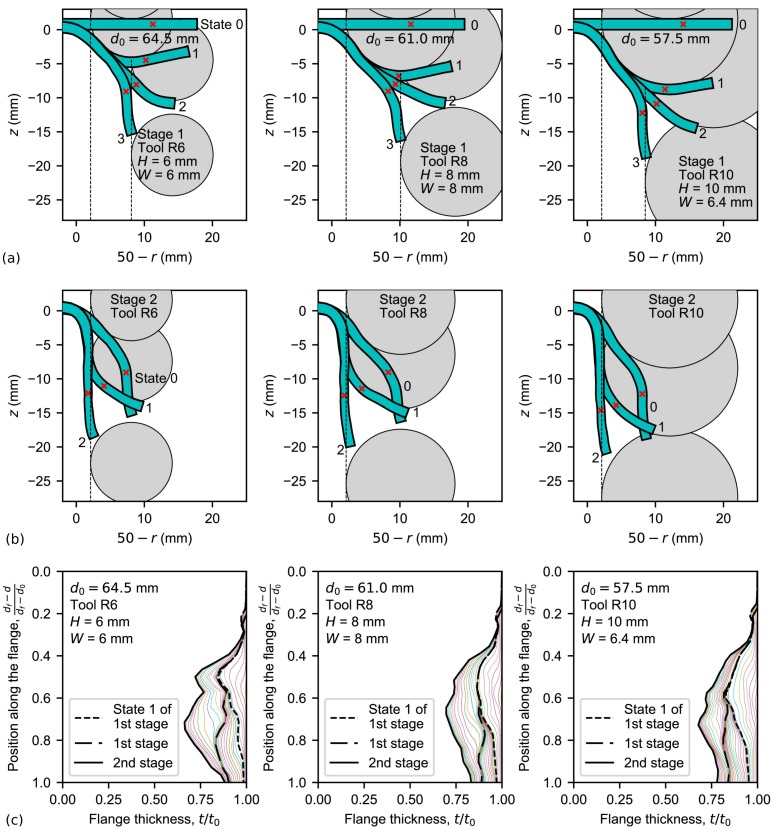
Simulations of hole-flanging by the proposed two-stage SPIF process: flange profile and tool position in (**a**) stage 1 and (**b**) stage 2; and (**c**) flange thinning.

The sheet profile evolutions and tool positions by the proposed two-stage SPIF process in both stages 1 and 2 are shown in Figure 12a,b, respectively. In stage 1, four simulation states are represented: (0) the initial state; (1) a tool step-down increment of H=R; (2) an intermediate tool position during the transition circular movement; and (3) the final state. In stage 2, three states are depicted: (0) the initial state whose sheet profile was obtained in stage 1; (1) an intermediate tool position; and (2) the final state. A red cross indicates again the position where maximum thinning occurs. Notice that this position is closer to the edge than those obtained for single-stage SPIF.

Figure 12a reveals the deformation mechanism inherent to the proposed two-stage SPIF process: the material resistance is being diminished by reducing the hole-flanged diameter to $d_{f}-2W$. Note that the flange section being bent in states 1–3 is similar for the three simulations. It should be remarked that simulation using a 10-mm tool radius and W=R=10 mm bent a very small zone of the edge and this led to a poorer thickness distribution. Instead, W=6.4 mm was set by locating the tool tip position just on the hole edge in state 1.

As deduced from Figure 12b, hole-flanging evolves in stage 2 by two consecutive reversal bending processes at states 1 and 2, respectively, to increase the inner diameter of the sheet part.

The thickness evolution is represented in Figure 12c. Thinning in state 1 of the first stage matches the one obtained in same state of the single-stage process, as expected. From states 1 to 3 of this stage, slight thinning occurs due to the edge bending process. In general, stage 2 distributes the thickness homogeneously along the flange.

### 4.4. Experimental Study

Hole-flanging by two-stage SPIF experimental tests was performed for evaluating the numerical analysis. The experiments were carried out using the same SPIF setup of the previous work for hole-flanging by single-stage SPIF [11]. A 6-mm tool radius was used to deform a 7075-O aluminium alloy of 1.6-mm thickness and 64.5-mm initial hole diameter. The parameter process was set to W=R=6 mm. The manufactured parts were cut along the flange and the resulting thickness profiles were measured by using an optical microscope. The test was performed twice leading to a similar thickness distribution, exhibiting differences lower than 2%.

**Figure 13:**
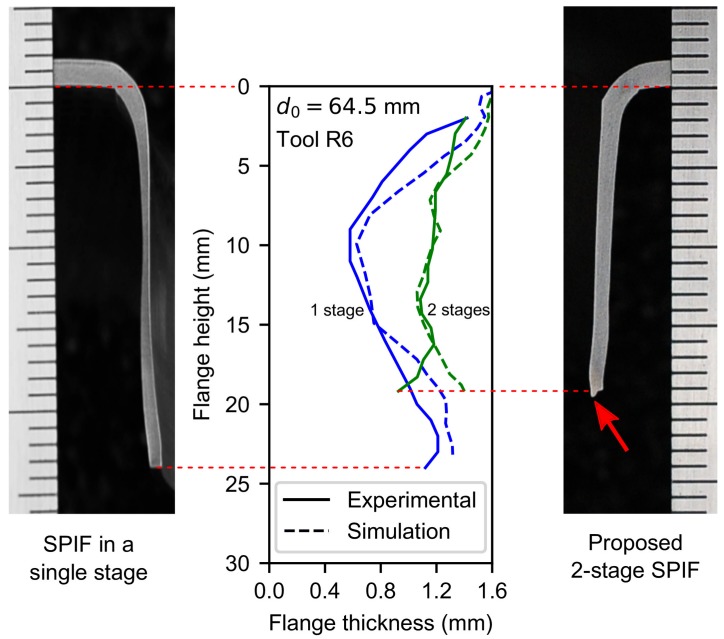
Views of hole-flange sections obtained by single-stage and two-stage SPIF experimental tests and comparison with numerical simulations.

Figure 13 compares the numerically predicted thickness profiles for both single- and two-stage SPIF operations with their experimental counterpart. As can be seen, the simulation of the two-stage process corresponds fairly to the thickness profile except at the edge zone. This slight divergence may be attributed to that the forming tool pressed up the hole edge during the last turns of its helical trajectory, producing a kind of burr at the edge which is highlighted with a red arrow in Figure 13. A small burr was also observed in specimens obtained by single-stage SPIF.

The maximum numerical deviations in terms of thickness with respect to the corresponding experimental results (see Figure 13) are about 10% in the critical zone for the single-stage SPIF process, whereas in the two-stage variant they do not exceed 5%. These results point out the ability of the proposed methodology to homogenize the thickness of the flange with a two-stage SPIF operation. Likewise, according to the numerical analysis presented here, significant improvements are also expected for the other two specimens analysed above ($d_{0}=61.0$ and 57.5 mm using tool radii R=8 and 10 mm, respectively). An experimental campaign is planned to assess the influence of tool radius on this methodology and will be discussed in a further publication.

## 5. Conclusions

An optimization procedure using two stages has been proposed, improving the thickness homogenisation along the flange as well as fabrication times in hole-flanging by SPIF. The methodology begins by analysing the single-stage process to understand the material deformation mechanism and failure. Accordingly, a customised two-stage SPIF strategy has been proposed and evaluated by FE simulations. Different tool radii and hole diameters in AA7075-O sheets have been considered.

The understanding of flange deformation during the single-stage variant has allowed defining a new improved two-stage SPIF strategy and reducing the process design times compared to trial-and-error methods. In this sense, the explicit analysis carried out in this work helped to perform FEA with reasonable computing times. The proposed two-stage SPIF process has also been corroborated experimentally.

The optimized two-stage strategy for hole-flanging by SPIF has significantly improved the thickness distribution and fabrication time compared to other multi-stage strategies found in the scientific literature. The forming tool radius *R* has played an important role to determine the intermediate part geometry that has been defined by parameters height *H* and width *W*. The higher the tool radius, the higher the parameter values to obtain the best thickness distribution. Indeed, good results in terms of homogeneous thickness have been obtained by using the simple rule H=W=R.

## Figures and Tables

**Table 1:** Mechanical properties for AA7075-O sheets.

*E* (GPa)	ν	YS (MPa)	*K* (MPa)	*n*
65.7	0.3	109.7	314	0.13

## Abbreviations

The following abbreviations are used in this manuscript:

SPIF	Single Point Incremental Forming
ISF	Incremental Sheet Forming
FLC	Forming Limit Curve (by necking)
FLD	Forming Limit Diagram
FFL	Fracture Forming Limit
FE	Finite Element
FEA	Finite Element Analysis
